# Study of CeO_2_ Modified AlNi Mixed Pillared Clays Supported Palladium Catalysts for Benzene Adsorption/Desorption-Catalytic Combustion

**DOI:** 10.3390/ma10080949

**Published:** 2017-08-15

**Authors:** Jingrong Li, Shufeng Zuo, Peng Yang, Chenze Qi

**Affiliations:** Zhejiang Key Laboratory of Alternative Technologies for Fine Chemicals Process, Shaoxing University, Shaoxing 312000, China; sfzuo@126.com (J.L.); yangpeng1898@163.com (P.Y.); qichenze@usx.edu.cn (C.Q.)

**Keywords:** AlNi-PILC, Pd-Ce, catalytic combustion, benzene, TPD/TPSR

## Abstract

A new functional AlNi-pillared clays (AlNi-PILC) with a large surface area and pore volume was synthesized. The performance of adsorption/desorption-catalytic combustion over CeO_2-_modified Pd/AlNi-PILC catalysts was also studied. The results showed that the *d*_001_-value and specific surface area (*S*_BET_) of AlNi-PILC reached 2.11 nm and 374.8 m^2^/g, respectively. The large *S*_BET_ and the *d*_001_-value improved the high capacity for benzene adsorption. Also, the strong interaction between PdCe mixed oxides and AlNi-PILC led to the high dispersion of PdO and CeO_2_ on the support, which was responsible for the high catalytic performance. Especially, 0.2% Pd/12.5% Ce/AlNi-PILC presented high performance for benzene combustion at 240 °C and high CO_2_ selectivity. Also, the combustion temperatures were lower compared to the desorption temperatures, which demonstrated that it could accomplish benzene combustion during the desorption process. Furthermore, its activity did not decrease after continuous reaction for 1000 h in dry air, and it also displayed good resistance to water and the chlorinated compound, making it a promising catalytic material for the elimination of volatile organic compounds.

## 1. Introduction

Volatile organic compounds (VOCs) have high vapor pressure and low water solubility at room temperature, and already have been recognized as major contributors to air pollution. They mainly come from industrial processes, fossil fuel combustion, cement concrete, and furniture coatings [1]. Among various VOCs, the carcinogenic benzene is one of the most abundant found in either industrial operations or at home [2]. It can bring photo-chemical smog, ozone generation, and offensive odors. The catalytic combustion method has been proved to be highly-efficient for VOCs degradation, providing carbon dioxide and water as final products (because of its higher efficiency, lower operating temperature, and less harmful by-products than thermal oxidation [3,4,5,6,7]).

The studies of catalysts for VOCs catalytic combustion have been reported, focusing on three types of catalysts based on noble metals [8,9], transition metal oxides [10,11] and rare earth metal oxides [10]. Generally, noble metal catalysts (Pt, Pd, and Au) [12,13,14] are commonly used for VOCs oxidation, and they usually represent higher activity than transition metal oxides. Particularly, supported Pd catalyst is one of the most used materials, due to its high activity for deep oxidation of VOCs at relatively low temperatures [15,16,17,18,19,20,21,22]. Moreover, as important promoters, rare earth elements (REE) with a special electronic structure have drawn much attention in recent years. REE can decrease the amount of noble metals, stabilize supports against thermal sintering, improve the performance of catalysts in storing/releasing oxygen, and reduce the reaction activation energy [23,24,25,26,27].

As is well known, the support is also an important factor for the performance of supported noble metal catalysts, and the choice of catalyst support usually depends on its specific surface area (*S*_BET_), pore size, and the capacity for interaction with metals. Generally, higher *S*_BET_ can provide more active sites and larger pore size more easily and allows the reactants to approach those catalytic active sites. Montmorillonite KSF (MMT) has been applied in initial clay due to its stable structure, low cost, and environmental compatibility [28,29,30,31]. Notably, as the modification of MMT, pillared clays (PILC) have a large *S*_BET_ and pore volume (*V*_P_), and the porous structure and physicochemical properties of MMT are improved significantly. In order to further improve the PILC performance, more attention has been directed toward PILC with mixed oxide pillars including Al-Zr, Al-Fe, and Al-Cr-PILC [6,9,32]. However, their applications are limited due to their poor thermal stability and durability. Some reports illustrate that various Ni-containing porous materials have good thermal and hydrothermal stability, such as Ni-Al-MCM-41 [33,34,35], Ni-zeolite [36], and Ni-Al-SBA-51 [37,38,39]. However, disadvantages still remain, including the complicated preparation process for supports. Therefore, there is an urgent need to simplify the procedures and synthesis of mixed oxide pillars containing Ni atoms.

Based on an understanding of the stability and synthesis of the PILC process, functional AlNi-PILC supports were prepared using a high temperature and high pressure hydrothermal method. Compared with MMT, AlNi-PILC displayed a larger specific surface area, a larger pore volume, and a high thermal stability. Therefore, it could be used as support to prepare the high performance catalyst. The influence of the introduction of CeO_2_ into Pd/AlNi-PILC for benzene combustion was also studied. The relationship between texture-structure and catalytic properties was systematically characterized and analyzed by X-ray diffraction (XRD), N_2_ adsorption/desorption, high resolution transmission electron microscopy and energy dispersive X-ray spectroscopy (HRTEM-EDS), the temperature-programmed desorption of benzene (benzene-TPD), and the in-situ temperature-programmed surface reaction of benzene (benzene-TPSR) experiments. The water and chlorobenzene were systematically studied in order to preliminarily explore the Pd/Ce/AlNi-PILC potential for further industrial application.

## 2. Experimental

### 2.1. Synthesis

MMT was used as initial material and the AlNi-pillaring agent was prepared using a hydrothermal method. The aqueous solution of Ni(NO_3_)_2_·6H_2_O and Locron L from Clariant (containing 6 mol/L Al ions) was mixed in autoclave (the molar ratio was Al/Ni = 5:1), and deionized water was added so that the concentration of Al ion was 2.0 mol/L. The autoclave was placed in an oven at 100 °C for 16 h and subsequently cooled down to 30 °C. The maintained solution was diluted to 600 mL and, finally, AlNi-pillaring agent was obtained. The following preparation of AlNi-PILC by the similar method was detailed in our previous research [6]. The *X*% Ce/AlNi-PILC samples were prepared by impregnation of Ce(NO_3_)_2_·6H_2_O (*X* = 2.5, 5, 7.5, 10, 12.5, and 15, respectively). After keeping impregnated samples at 30 °C for 12 h, the samples were dried at 110 °C and subsequently calcined at 400 °C for 2 h. The Pd/*X*% Ce/AlNi-PILC samples were obtained by impregnating *X*% Ce/AlNi-PILC with an aqueous H_2_PdCl_4_ solution at 30 °C for 12 h, and the yellow was completely disappeared under an infrared lamp. Then, 5% hydrazine hydrate was added and reacted for 3 h, and the samples were filtered and washed by deionized water until no Cl^−^ was detected in the filtrate by aqueous AgNO_3_ solution. Samples were dried at 110 °C, and subsequently calcined at 400 °C for 2 h. The Pd content of all catalysts was 0.2 wt. %.

### 2.2. Catalytic Activity Tests

The experiments were performed with a 350 mg catalyst in a WFS-3010 microreactor (Xianquan, Tianjin, China). An analysis of the reactants and products was performed by on line gas chromatography (Shimadzu, GC-14C, Kyoto, Japan) with a flame ionization detector (FID). The reactive flow (120 mL/min) was composed of gaseous benzene (1000 ppm) in dry air with a gas hourly space velocity (GHSV) of 20,000 h^−1^. The data were recorded and analyzed using an N2000 chromatography data workstation. The catalytic activity was determined by parallel analytical measurement at a certain temperature (parallel determination of three identical catalysts, approximately 0.5 h per series), and the average was taken as the final conversion. And the benzene conversion was calculated as follows: benzene conversion (%) = $\frac{[\mathrm{benzene}]\mathrm{in}-[\mathrm{benzene}]\mathrm{out}}{[\mathrm{benzene}]\mathrm{in}}\times 100\%$ (where [benzene]_in_ is the benzene concentration in the feed gas, and [benzene]_out_ is the benzene concentration in the products).

In order to study the “mixture effect” of the feed gas, 100 ppm chlorobenzene and 10,000 ppm water vapor were introduced, respectively. The any possible combustion products were further detected by mass spectrometry (MS, QGA, Hiden, Warrington, UK). H2O and CO_2_ were the only detected byproducts, and thus conversion was calculated based on benzene consumption. The durability of catalysts for benzene combustion was also investigated under the same condition.

### 2.3. Characterization

The samples were characterized by the XRD technique (PANalytical, Almelo, The Netherlands) for the *d*_001_ value and phase composition. The specific surface area (*S*_BET_), mesoporous area (*A*_mes_), total pore volume (*V*_p_), micropore volume (*V*_mic_), and pore size distribution of the samples were determined by N_2_ adsorption isotherms. High-resolution transmission electron microscopy (HRTEM, JEOL, Valley, Japan) was employed to get the catalyst morphology and particle size. The chemical compositions of the catalysts were determined with energy dispersive X-ray spectroscopy (EDS, JEOL, Valley, Japan). All the characterization methods for the samples have been reported and detailed in our previous research [3,9,10]. The palladium and Ce contents were measured by an Inductively Coupled Plasma Optical Emission Spectrometer (ICP-OES, Leeman Labs, Hudson, NH, USA) after the dissolution of the catalysts in a mixture of HF and HNO_3_ solution. Benzene-TPD and the benzene-TPSR) experiments were performed in a quartz tube. Prior to adsorption of benzene, the catalyst (350 mg) was pretreated in dry air at 300 °C for 0.5 h. After being cooled down to 50 °C, the adsorption of benzene was carried out under a flow of N_2_/benzene (TPD) or (20%O_2_/Ar) /benzene (TPSR) until adsorption saturation, as indicated by the stable signal of benzene in the mass spectrometer. Then, a pure N_2_ flow was carried out for 1h to clean the benzene in the pipe. Finally, the desorption or oxidation of benzene was implemented followed by a flow of pure N_2_ (TPD) or (20%O_2_/Ar)/benzene (TPSR) by a step of 7.5 °C/min from 50 to 500 °C. The concentration of benzene and the final products (CO*_x_* and H_2_O) were measured on-line by MS.

## 3. Results and Discussion

### 3.1. Catalytic Performance and Stability Test

**Figure 1:**
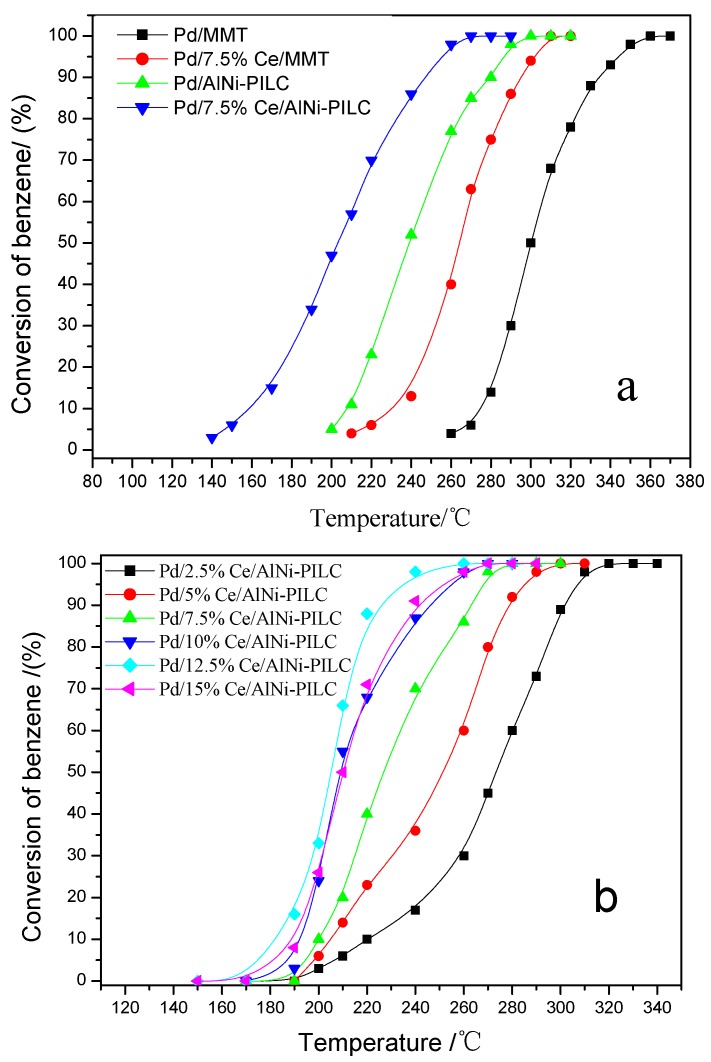
(**a**) Influence of the addition of Ce into Pd/MMT and Pd/AlNi-PILC for benzene complete oxidation; (**b**) influence of the content of Ce into Pd/AlNi-PILC for benzene complete oxidation. Benzene concentration: 1000 ppm; gas hourly space velocity (GHSV): 20,000 h^−1^; Catalyst amount: 350 mg.

Generally, benzene is completely degraded at 600 °C under a no catalysts condition. The catalytic activity of catalysts for benzene combustion is displayed in Figure 1a. It can be seen that Pd/MMT exhibits poor performance and the complete conversion of benzene does not occur until 350 °C. Pd/AlNi-PILC is able to completely degrade benzene at 318 °C. The results suggest that AlNi-PILC is more suitable to be a catalytic support. In addition, Ce doping significantly improved the catalytic activities of Pd/MMT and Pd/AlNi-PILC. Therefore, the effect of Ce content was also investigated in Figure 1b. According to the values of *T*_98%_ (temperature which benzene conversion reaches 98%), the order for the catalytic activity is Pd/AlNi-PILC (318 °C) < Pd/2.5% Ce/AlNi-PILC (310 °C) < Pd/5% Ce/AlNi-PILC (290 °C) < Pd/7.5% Ce/AlNi-PILC (270 °C) < Pd/10% Ce/AlNi-PILC (260 °C) < Pd/15% Ce/AlNi-PILC (255 °C) < Pd/12.5% Ce/AlNi-PILC (240 °C). The results further demonstrate that the addition of various amounts of Ce improves the catalytic activities of the Pd/AlNi-PILC catalysts. When Ce loading is <12.5%, the activity of the catalyst increases with the addition of Ce content. However, the catalytic activity decreases when Ce loading is 15%, which is due to the fact that Ce acts as a promoting component and too much Ce loading may override the PdO active sites. Similar results have been reported in our previous research [40,41,42]. Thus, proper Ce content and the metal-metal interaction can significantly enhance the Pd/Ce/AlNi-PILC activity. Particularly, the catalyst of 12.5% Ce loading exhibits the highest activity and the *T*_98%_ of benzene conversion is 240 °C. In addition, the catalytic combustion performances of benzene over some typical noble metal-based catalysts [12,15,43,44,45] were listed in Table S1, and Pd/12.5% Ce/AlNi-PILC catalyst possesses better performance.

**Figure 2:**
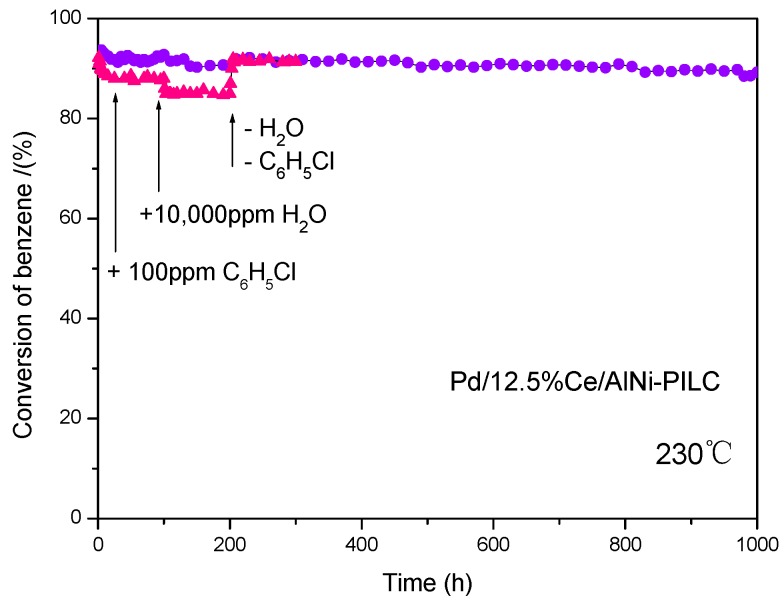
Lifetime test performed for Pd/12.5% Ce/AlNi-PILC at 230 °C.

Figure 2 presents the lifetime test result for the most active catalyst (Pd/12.5% Ce/AlNi-PILC) at 230 °C for 1000 h. The conversion of benzene remained at around 94% and no obvious deactivation was observed, which demonstrates that it exhibits stable catalytic activity for combustion. Moreover, in practice, water and chlorinated VOCs always exist in the waste gases. As shown in Figure 2, in the first continuous 100 h reaction in the presence of 100 ppm chlorobenzene, the activity of the catalyst decreases slightly, because of the effect of competitive adsorption and oxidation on the active sites. When 10,000 ppm water is introduced, its activity further decreases, due to the competitive adsorption effect. Interestingly, the catalytic activity is recovered to the initial level after water and chlorobenzene are removed. In all, these results mentioned above confirm that Pd/12.5% Ce/AlNi-PILC presents a wide range of possibilities for further industrial application, due to its high catalytic performances for combustion of both non-chlorinated VOCs and chlorinated VOCs, as well as its good resistance to water.

### 3.2. Effects of Loading Pd Content and Benzene Concentration

Figure S1 presents the effects of Pd loading contents on the benzene catalytic performance of *X*% Pd/12.5% Ce/AlNi-PILC. Benzene conversion increases with the increase of Pd content from 0.2 to 0.5%. When the content of Pd increases to 0.5%, the activity changes slightly compared to 0.4% Pd catalyst. It suggests that the addition of proper Pd content can present high dispersion on support and improve the catalytic performances obviously. Figure S2 presents the effects of benzene inlet concentrations (500 to 2500 ppm) on the catalytic performance of 0.2% Pd/12.5% Ce/AlNi-PILC. Benzene conversion increases appreciably with the increase of its inlet concentrations from 500 to 1500 ppm. When low concentration benzene is fed, the amount of chemisorbed benzene on catalyst active sites is low and can be a reaction controlling factor. However, chemisorbed oxygen on the catalyst active sites can become the reaction controlling factor when the benzene concentration increases to a certain point, thus the conversion of benzene should be prohibited.

### 3.3. XRD and N_2_ Adsorption Analysis

**Figure 3:**
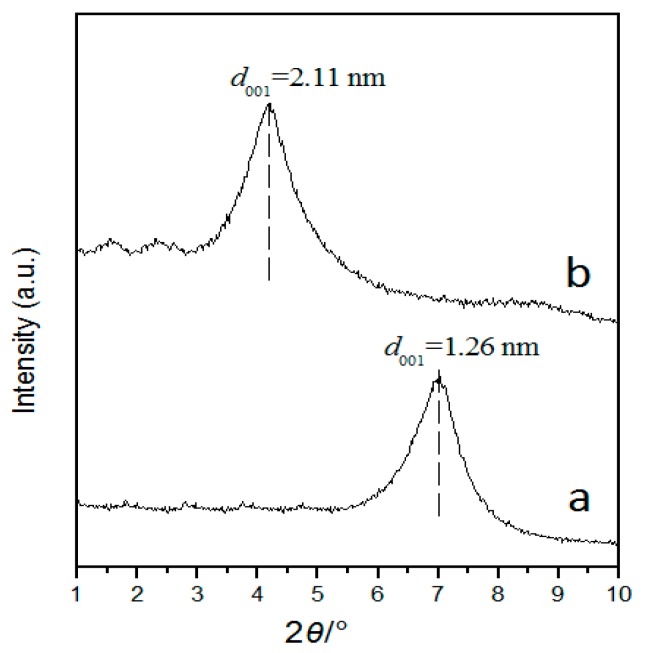
Small-angle X-ray diffraction (XRD) patterns. (**a**) Montmorillonite (MMT); (**b**) AlNi-PILC.

The small-angle XRD patterns are obtained to the confirm two-dimensional layered structure of MMT and AlNi-PILC in Figure 3. It can be seen that the *d*_001_ spacing of MMT and AlNi-PILC is 1.26 nm and 2.11 nm, respectively, with corresponding 2*θ* values of 6.99° and 4.18°. It suggests that AlNi poly-cations intercalated between the layers are much larger than Na ions in size, leading to a layered structure with large spacing. Thus, the synthesis of pillared clays is successful.

**Figure 4:**
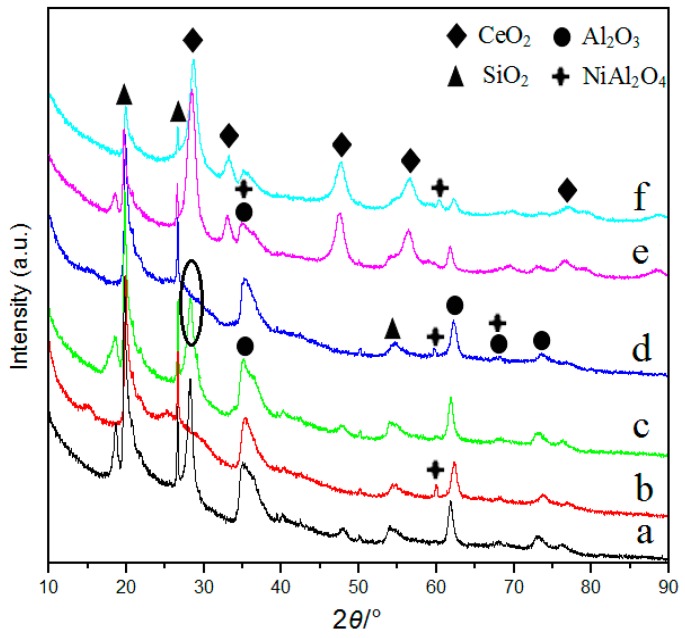
Long-angle XRD patterns. (**a**) MMT; (**b**) AlNi-PILC; (**c**) Pd/MMT; (**d**) Pd/AlNi-PILC; (**e**) Pd/12.5% Ce/MMT; (**f**) Pd/12.5% Ce/AlNi-PILC.

Figure 4 shows the high-angle XRD patterns of samples. Both MMT and AlNi-PILC contain similar cristobalite peaks (23.3°) and quartz peaks (26.5°), which are the characteristic peaks of montmorillonite. It suggests that pore structure is well preserved during the synthesis of AlNi-PILC. It must also be mentioned that a Al-Si polymeride peak (28.14°) disappears when NiAl_2_O_4_ peaks are formed by the interaction between nickel oxide and alumina during the calcination process. These peaks appear at 37.0°, 59.7°, and 64.5°, respectively, and most were overlapped with the peaks of Al_2_O_3_ [46]. Thus, some feature diffraction peaks of Al_2_O_3_ became weaker. Studies [46] have shown that spinal NiAl_2_O_4_ also promotes Pd catalysts to be more resistant to deactivation, which has been proved in the above lifetime test (Figure 2). In addition, neither palladium nor palladium oxide is detected for catalysts containing Pd. The result shows that the PdO particles were small, which is below the X-ray detection range, or Pd was highly dispersed on the supports. Notably, for the Pd/12.5% Ce/AlNi-PILC catalyst, the intensity of CeO_2_ peaks becomes significantly weaker than Pd/12.5% Ce/MMT, revealing higher dispersion of CeO_2_ on AlNi-PILC. From the catalytic activity and XRD results, it can be observed that better dispersion of CeO_2_ on AlNi-PILC is one of the key factors to enhance the catalytic activity of the catalysts. The above results also indicate that AlNi-PILC is useful for the high dispersion of PdO and CeO_2_ on its surface, which is further confirmed by below HRTEM images.

**Figure 5:**
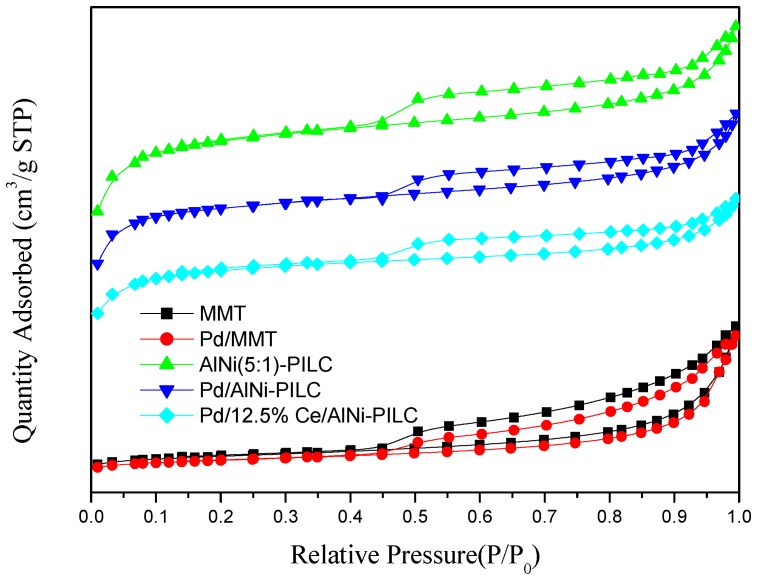
N_2_ adsorption/desorption isotherms of the samples.

The textural properties of all samples were characterized by the N_2_ adsorption/desorption method (Figure 5). Textural properties are listed in Table 1, and it can be seen that all samples provided the IV type isotherm of the miro-mesoporous materials with a sharp ramp in the relative pressure of 0.45, which is due to the capillary condensation of nitrogen in pores [47]. Additionally, *S*_BET_, *A*_mes_, and *V*_mic_ of all samples are summarized in Table 1. The *S*_BET_ of AlNi-PILC reaches to 374.8 m^2^/g, which is much larger than MMT. In addition, the *S*_BET_ of two synthesized samples (Pd/AlNi-PILC and Pd/12.5% Ce/AlNi-PILC) decreases to 298.4 m^2^/g and 230.5 m^2^/g, respectively, in which doped cations may enter the micro-mesopores of AlNi-PILC. Some similar results about the incorporation of other metals (such as Co, Nd, La, etc.) into the silicate framework are reported [48,49]. It is important to highlight that the values of *S*_BET_ are not connected with the order for activity of benzene combustion, revealing that it is not the only factor affecting catalytic oxidative performance.

Table 1 also shows that all samples possess different *V*_mic_ of 0.0018–0.1336 cm^3^/g and the *V*_p_ of 0.054 to 0.212 cm^3^/g, respectively. The large *S*_BET_ (374.8 m^2^/g) and *V*_P_ (0.212 cm^3^/g) of the AlNi-PILC are responsible for the well dispersion of the metallic particles and exposing more metallic sites on the surface for catalytic application. This is supported by the higher catalytic activity of the Pd/12.5% Ce/AlNi-PILC catalyst (Figure 1 and Table 1). In conclusion, the textural characterization results suggest that some Pd^2+^ and Ce^4+^ reach the inner porous network of AlNi-PILC, resulting in a strong interaction among PdO, CeO_2_, and AlNi-PILC.

### 3.4. HRTEM Analysis

**Figure 6:**
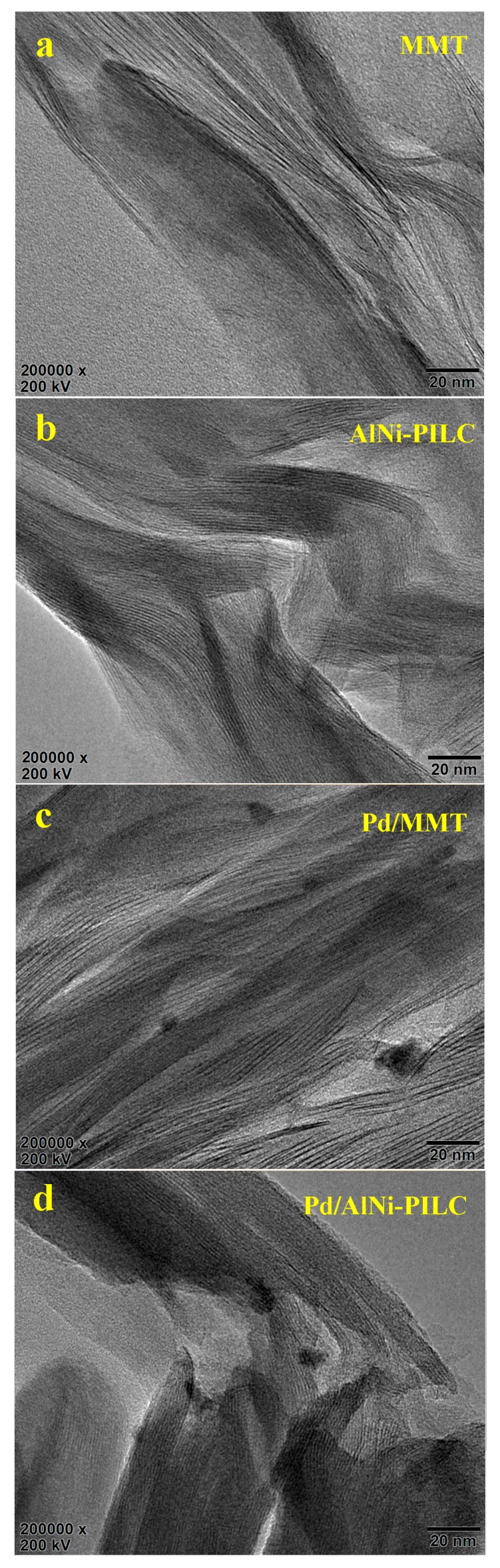

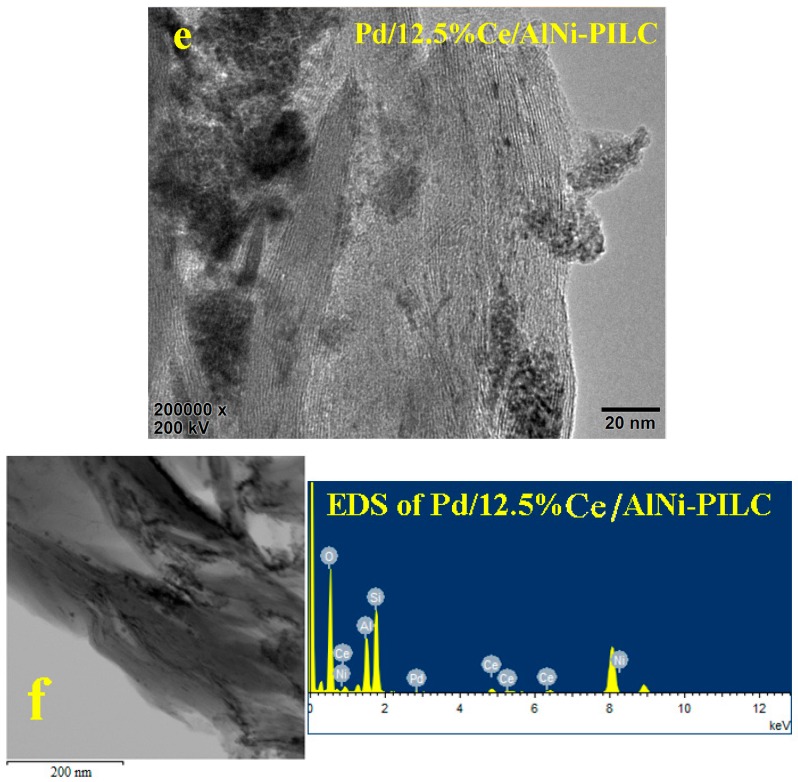
High resolution transmission electron microscopy (HRTEM) pictures. (**a**) MMT; (**b**) AlNi-PILC; (**c**) Pd/MMT; (**d**) Pd/AlNi-PILC; (**e**) Pd/12.5% Ce/AlNi-PILC; (**f**) the energy dispersive X-ray spectroscopy (EDS) spectra.

Figure 6 presents HRTEM pictures and the EDS spectra of MMT, Pd/MMT, Pd/AlNi-PILC, and Pd/12.5% Ce/AlNi-PILC. The HRTEM image of AlNi-PILC shows that the material has a better-ordered hexagonal arrays structure than MMT. HRTEM and the Map data picture of Pd/MMT show that PdO particles have more serious aggregation than Pd/AlNi-PILC. Also, the incorporation of CeO_2_ to the Pd catalyst leads to a higher PdO dispersion than that of Pd/AlNi-PILC, which is the result of the interaction with CeO_2_ and PdO. For the Pd/12.5% Ce/AlNi-PILC catalyst, in spite of its high Ce concentration, well dispersed PdO and CeO_2_ nanoparticles are obtained, which is consistent with the XRD result. In addition, after the addition of Ce and Pd, the particle sizes of AlNi-PILC do not change significantly and still present in an ordered framework. The Map data image of Pd/12.5% Ce/AlNi-PILC clearly reveals the well-dispersed active ingredients on AlNi-PILC. Pd and Ce were identified in the EDS spectra, confirming the successful loading of the active ingredients on the surface of AlNi-PILC. On the basis of the above results, we conclude that a higher dispersion of PdO-CeO_2_ on AlNi-PILC may be responsible for the good activity of these samples.

### 3.5. ICP-OES Analysis

The nominal mass percentage of Pd for all the catalysts is 0.2 and the Ce for Pd/12.5% Ce/AlNi-PILC is 12.5%, respectively. The real metal loadings of different catalysts measured by ICP-OES are listed in Table S2. The real concentration of Pd in all the catalysts is about 0.19%. In the fresh Pd/12.5% Ce/AlNi-PILC catalyst, the real contents of Pd and Ce are 0.187% and 12.0%, respectively, and no significant variation is observed after reaction. This indicates that AlNi-PILC can stabilize the active phase, and that this catalyst is reusable in benzene combustion and can perform well in a wide range of applications for the combustion of VOC.

### 3.6. TPD Analysis

**Figure 7:**
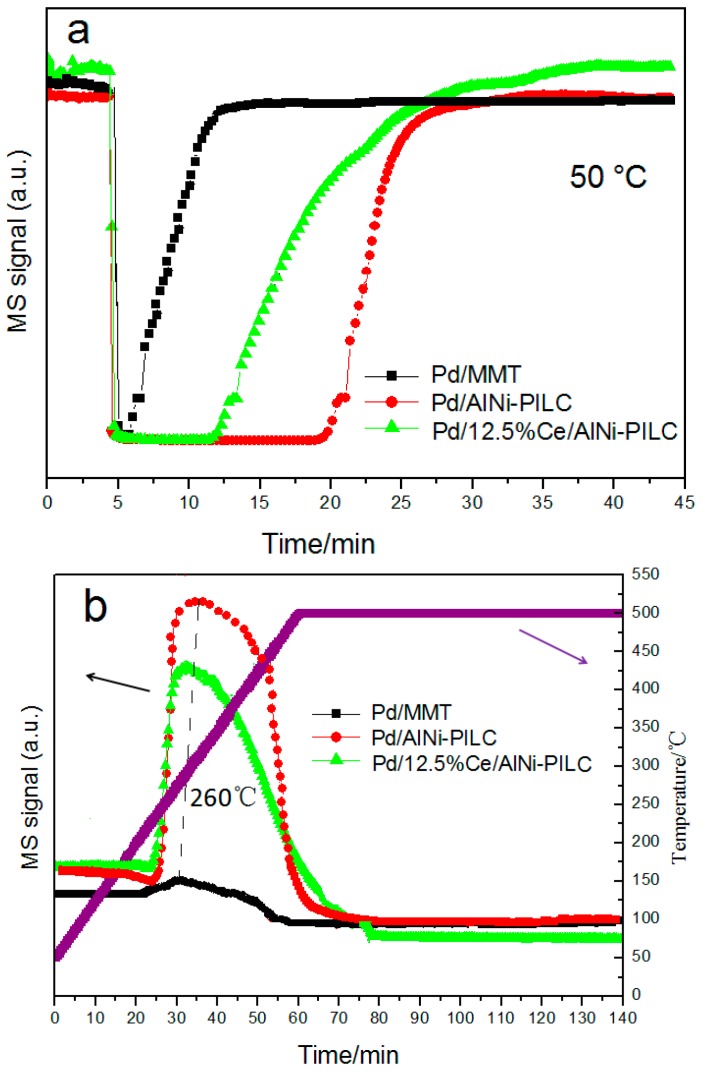
(**a**) Benzene (*m*/*z* = 78) adsorption profiles in temperature-programmed desorption (TPD) over Pd/AlNi-PILC, Pd/12.5% Ce-AlNi-PILC, and Pd/MMT catalysts; (**b**) benzene (*m*/*z* = 78) desorption profiles in TPD over Pd/AlNi-PILC, Pd/12.5% Ce-AlNi-PILC, and Pd/MMT catalysts.

Figure 7a shows the adsorption of benzene (*m*/*z* = 78) profiles of the catalysts in the TPD test. Compared with the MMT catalyst, the amount of benzene adsorption increases sharply when AlNi-PILC is used as support, and the order is Pd/AlNi-PILC > Pd/12.5% Ce/AlNi-PILC > Pd/MMT. This is probably because the large interlayer distance and pore volume of AlNi-PILC is advantageous for the adsorption of benzene. By integrating over the adsorption peaks, the benzene adsorption capacities of Pd/MMT, Pd/AlNi-PILC, and Pd/12.5% Ce/AlNi-PILC are calculated to be about 11.0, 28.5, and 36.8 μmol/g, respectively. Compared to the Pd/AlNi-PILC catalyst, the amount of benzene adsorption is decreased over Pd/12.5% Ce/AlNi-PILC, which may be caused by the decrease in specific surface area and the pore volume. Figure 7b presents the Pd/12.5% Ce/AlNi-PILC that exhibits a high desorption temperature, which leads to a high adsorption strength and catalytic activity. In addition, the desorption temperature for the reactant over the catalysts should have a great influence on the catalytic activity of the catalysts. Generally, the closer the desorption temperatures of benzene and O_2_ to the combustion temperature of benzene, the higher benzene conversion will be achieved. The conclusion will be further investigated, as shown below.

### 3.7. Benzene-TPSR Analysis

As is well known, the catalytic process is a dynamic and in-situ surface reaction. Thus, in order to investigate the oxidative performances of the catalysts under the dynamic condition and get more information on the real oxidation process, as well as the adsorption/desorption and oxidizing properties of the catalysts for benzene combustion, the evolution of any possible organic byproducts and the final products (CO*_x_* and H_2_O) on the catalyst surface are evaluated by the in-situ TPSR technique [11].

**Figure 8:**
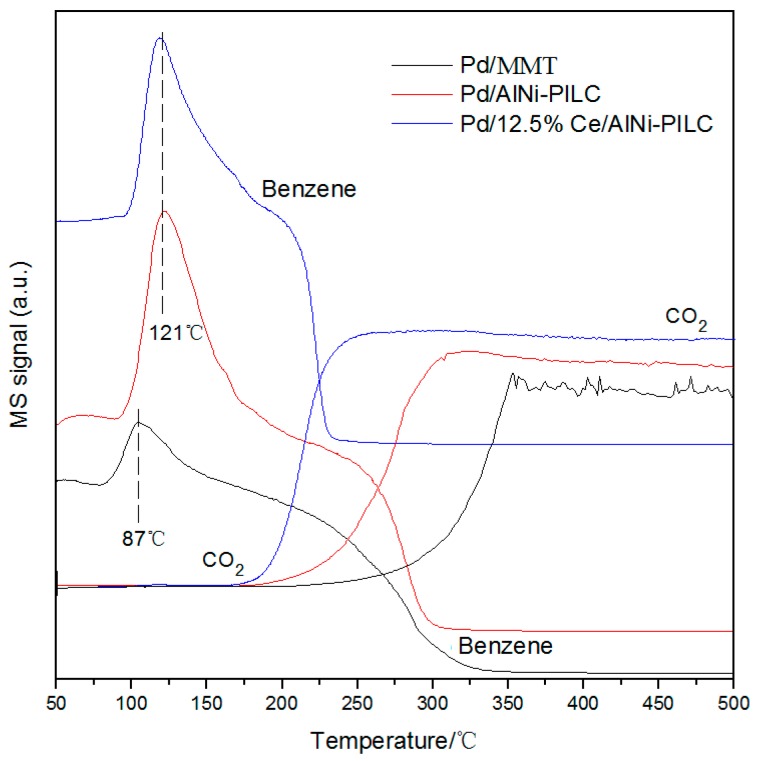
Results of benzene-TPSR characterization for benzene combustion over the catalysts.

As shown in Figure 8, in the range of 50 °C to 200 °C, the signal of benzene in the Pd/12.5% Ce/AlNi-PILC catalyst is observed while the signal of CO_2_ is absent, indicating that only the desorption of benzene occurs. Compared with the TPD results, the peak temperature of benzene over three catalysts is shifted to a lower temperature in the presence of gas phase O_2_, and the temperature for desorption of the benzene signal decreases in the order of Pd/12.5% Ce/AlNi-PILC ≈ Pd/AlNi-PILC > Pd/MMT. The results reveal that the strong interaction between AlNi-PILC and metals enhances benzene adsorption.

In addition, the desorption peak of benzene over Pd/MMT is smaller than Pd/12.5% Ce/AlNi-PILC, implying that its adsorption capacity is lower, which is not beneficial for the benzene oxidation reaction. As the reaction temperature increases, the oxidation of benzene gradually becomes obvious, due to the detection of CO_2_. The final products (CO*_x_* and H_2_O) were measured on–line by MS and the result indicates that CO_2_ is the only carbon product. It indicates that the above catalysts have high selectivity and high activity. Moreover, the temperature for the disappearance of the benzene signal increase is in the order of Pd/12.5% Ce/AlNi-PILC (240 °C) < Pd/AlNi-PILC (315 °C) < Pd/MMT (350 °C), and the temperature for the appearance of the CO_2_ signal increase is consistent with the above order. It is noteworthy that for Pd/12.5% Ce/AlNi-PILC, the degradation temperature (240 °C) of benzene is lower than the desorption temperature (260 °C), which helps complete benzene combustion during the desorption process, so it exhibits high catalytic activity.

## 4. Conclusions

In this work, AlNi-PILC material and Pd/Ce/AlNi-PILC catalysts with different Ce content were successfully synthesized and used in the catalytic combustion of low concentration benzene. The structure and redox properties of these materials were characterized by XRD, N_2_ adsorption, HRTEM-EDS, TPD, and TPSR techniques. XRD and N_2_ adsorption results indicate that AlNi-PILC material shows higher ordered hexagonal pore structure and higher *S*_BET_ than MMT. Also, Pd-Ce-supported catalysts still maintain ordered layer structures. From the HRTEM-EDS results, the incorporation of CeO_2_ to the Pd catalysts leads to a higher dispersion than that of Pd/AlNi-PILC. The appropriate crystallized size of AlNi-PILC support and the high dispersed PdO nanosize particles might have a large significance for Pd/12.5% Ce/AlNi-PILC stability and catalytic activity. The TPD and TPSR results show that the Pd/12.5% Ce/AlNi-PILC high capacity for adsorption/desorption-catalytic combustion of benzene are due to the high benzene adsorption strength and the similar temperature between benzene desorption and the combustion process. Therefore, Pd/12.5% Ce/AlNi-PILC can complete benzene combustion at 240 °C. Furthermore, stability tests indicate that there is no obvious deactivation for the Pd/12.5% Ce/AlNi-PILC catalyst in the 1000 h continuous reaction, whether in the water condition or in the presence of C_7_H_8_, which indicates that it deserves more attention and that there is potential for industrial application.

## Figures and Tables

**Table 1:** Characteristics of the samples: surface area and pore volume.

Samples	*S*_BET_^a^ (m^2^/g)	*A*_mes_^b^ (m^2^/g)	*V*_p_^c^ (cm^3^/g)	*V*_mic_^d^ (cm^3^/g)
MMT	24.6	20.1$	0.054	0.0018
AlNi-PILC	374.8	86.1	0.212	0.1336
Pd/MMT	20	17.9	0.05	0.0007
Pd/AlNi-PILC	298.4	61.4	0.17	0.1101
Pd/12.5% Ce/AlNi-PILC	230.5	56.8	0.131	0.0804

^a^ BET specific surface area; ^b^ Calculated from BJH method; ^c^ Total pore volume estimated at P/P_0_ = 0.99; ^d^ Calculated from the t-method.

## Supplementary Materials

The following are available online at www.mdpi.com/2072-6651/9/8/949/s1, Figure S1: Effects of Pd content on catalytic activity of Pd/12.5% Ce/AlNi-PILC for benzene combustion. Benzene concentration: 1000 ppm; GHSV: 20,000 h^−1^; Catalyst amount: 350 mg. Figure S2: Effects of inlet concentration on benzene catalytic combustion over Pd/12.5% Ce/AlNi-PILC. Benzene concentration: 500–2500 ppm; GHSV: 20,000 h^−1^; Catalyst amount: 350 mg. Table S1: Main data of reported literatures on catalytic combustion of benzene over supported noble metal catalysts. Table S2: Metal loadings (wt. %) of different catalysts.
