# Supplementary material for: Study of CeO2 Modified AlNi Mixed Pillared Clays Supported Palladium Catalysts for Benzene Adsorption/Desorption-Catalytic Combustion
Source: Materials (Basel). 2017 Aug 15;10(8):949. doi: 10.3390/ma10080949 (PMC5578315; doi:10.3390/ma10080949)
Supplement: Supplementary file 1 [file materials-10-00949-s001.pdf]

Supplementary Materials: Study of CeO_2 Modified AlNi Mixed Pillared Clays Supported Palladium Catalysts for Benzene Adsorption/Desorption-Catalytic Combustion

Table S1. Main data of reported literatures on catalytic combustion of benzene over supported noble metal catalysts.

**Catalysts**	**Reaction conditions**			T_{100} **(℃)**
	**Active sites (wt.%)**	**GHSV (h^{-1})**	**Benzene**	
Pd-Ni/SBA-15	0.16%Pd	120,000	1000 ppm	260
Pd/La/ZSM-5	0.2%Pd-6%La	20,000	1000 ppm	290
Pd/γ-Al_2O_3	0.4%Pd	30,000	1000 ppm	370
Pt/C	3% Pt	8,000	1000 ppm	300
Pd–Co_3/AlO	1.0%Pd	30,000	1000 ppm	330
**Pd/Ce/AlNi-PILC [our work]**	**0.2% Pd-12.5% Ce**	**20,000**	**1000 ppm**	**240**

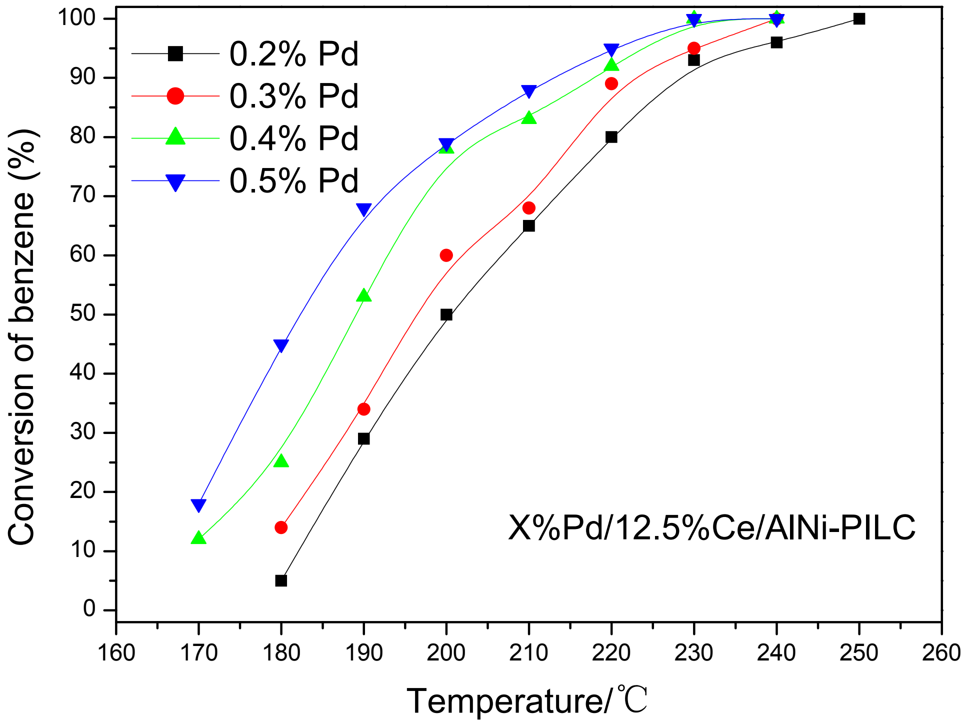

Figure S1. Effects of Pd content on catalytic activity of Pd/12.5% Ce/AlNi-PILC for benzene combustion. Benzene concentration: 1000 ppm; GHSV: 20,000 h^{-1}; Catalyst amount: 350 mg.

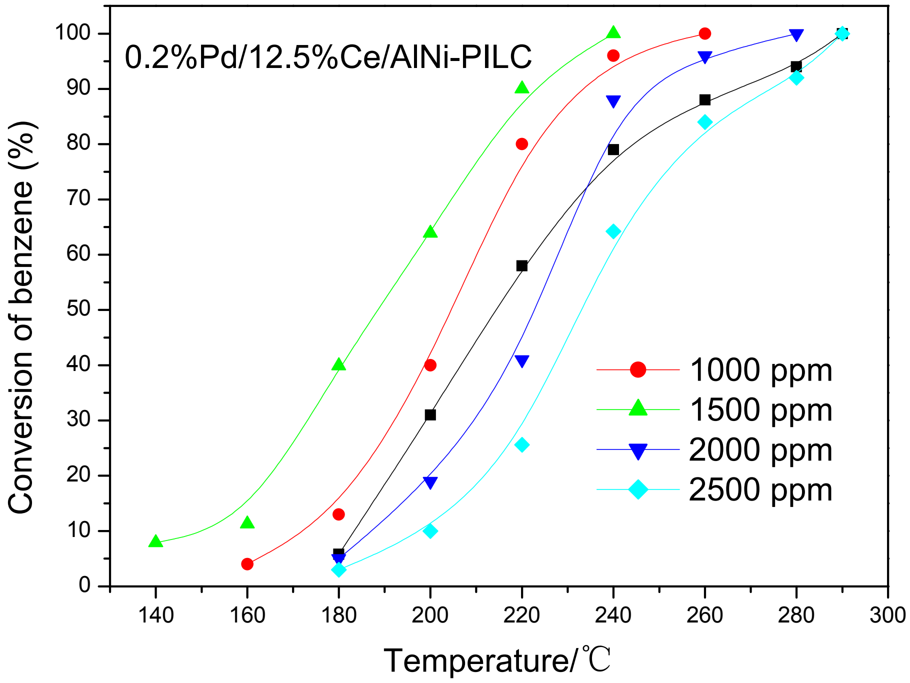

Figure S2. Effects of inlet concentration on benzene catalytic combustion over Pd/12.5% Ce/AlNi-PILC. Benzene concentration: 500-2500 ppm; GHSV: 20,000 h^{-1}; Catalyst amount: 350 mg.

Table S2. Metal loadings (wt.%) of different catalysts

Catalysts	Pd [wt.%]	Ce [wt.%]
Pd/MMT	0.186	--
Pd/AlNi-PILC	0.192	--
Pd/12.5% Ce/AlNi-PILC (fresh)	0.187	12.0
Pd/12.5% Ce/AlNi-PILC (used) [a]	0. 185	12.3

[a] The reused catalyst after reaction for 3 times.
